# A Descriptive Cross-Sectional Study on Clinical Epidemiology of Different Types of Cancer in a Tertiary Care Centre: Insights From Eastern India

**DOI:** 10.7759/cureus.62529

**Published:** 2024-06-17

**Authors:** Ajit K Kushwaha, Nalini Kumari, Soni kumari, Vijay M Motghare, Sumana Sen, Mukesh K Niraj, Muklesh K Mehta

**Affiliations:** 1 Surgical Oncology, Rajendra Institute of Medical Sciences, Ranchi, IND; 2 Pharmacology, Apollo Institute of Medical Sciences and Research, Hyderabad, IND; 3 Pharmacology, Laxmichandravansi Medical College, Garhwa, IND; 4 Pharmacology, Government Medical College, Nagpur, IND; 5 Biochemistry, Rajendra Institute of Medical Sciences, Ranchi, IND; 6 General Surgery, Rajendra Institute of Medical Sciences, Ranchi, IND

**Keywords:** epidemiology, eastern india, prevalence, statistics, cancer

## Abstract

Background and aim

Cancer poses a significant burden in India, with a considerable number of people living with the disease and a substantial increase in new cases every year. Hence, considering the unique challenges faced by developing nations regarding the disease burden, this study has been designed. The aim of this work was to carry out a descriptive retrospective cross-sectional study on various types of cancer conducted in a tertiary care centre in India.

Methods

One thousand cancer patients who attended the outpatient department (OPD) from tertiary care cancer hospitals from July 2019 to December 2023 in Eastern India were enrolled. Patients included were of either gender, with their demographic details and the disease duration, who visited the OPD of hospitals meeting the eligibility criteria. Exclusion criteria were terminally ill cancer patients and patients who did not visit the outpatient department of the studied site. Descriptive analysis and chi-square test were carried out using the SPSS statistical software, version 20.0 (IBM Corp., Armonk, NY) for data analysis. Ethics committee approval was taken.

Results

Gastrointestinal tract cancer (31.3%, n=313) and breast cancer (19.8%, n=198) were found to be the most common types of cancer among all. Out of the total patients studied, 41.1% were males and 58.9% were females. Among regions, North Chotanagpur had the highest (40.5%) prevalence, followed by South Chotanagpur (26.0%). The majority of individuals belonged to 41 to 60 years (49.0%, n=490), followed by 21-40 years (28.9%, n=289). Gastrointestinal cancer was more prevalent among males (35.5%, n=146), while breast cancer was predominant among females (31.4%, n=185).

Conclusion

Cancer is more prevalent among rural females (58.9%), providing valuable insights into the prevalence of various cancers and highlighting differences between regions, age groups, and genders.

## Introduction

Cancer encompasses a diverse group of diseases characterized by the uncontrolled growth of abnormal cells. These cells often infiltrate adjacent tissues or spread to other parts of the body, a process known as metastasis [1,2]. Cancer is among the leading causes of death worldwide; in 2022, there were almost 20 million new cases and 9.7 million cancer-related deaths worldwide. In 2022, India saw an estimated 1,461,427 new cancer cases, translating to a crude rate of 100.4 per 100,000 people. One in nine Indians is expected to develop cancer during their lifetime. Among males, lung cancer was the most common type, while breast cancer was the most prevalent among females. For children aged 0-14, lymphoid leukemia was the leading cancer, representing 29.2% of cases in boys and 24.2% in girls. The incidence of cancer cases is projected to increase by 12.8% by 2025 compared to 2020. By 2040, the number of new cancer cases per year is expected to rise to 29.9 million and the number of cancer-related deaths to 15.3 million.

Epidemiology is defined as the study of disease frequency, distribution, and determinants of diseases and other health-related conditions within human populations, with its application aimed at promoting health and preventing and controlling health problems. This field mainly focuses on investigating the distribution and determinants of disease occurrence among population groups rather than individuals, as well as cross-sectional studies, a type of transversal descriptive and observational epidemiological study conducted at a single point in time without a follow-up period [3-5].

Cancer surveillance plays a crucial role in epidemiology and public health by providing essential data insights into the prevalence of different cancer types within specific populations. It serves as a foundation for evidence-based health interventions that effectively combat cancer. Furthermore, it encompasses monitoring various health outcomes among cancer patients, such as survival rates following diagnosis and treatment. These efforts not only contribute to the field of epidemiology but also inform the development of comprehensive strategies for addressing cancer at a broader level [4-6]. According to data, approximately half of all patients diagnosed with cancer can presently be cured using existing treatment options, while the other half unfortunately do not survive the disease. The World Health Organization (WHO) highlights that around 40% of global cancer cases could be avoided through timely screening and intervention measures. It gives or adds a considerable global burden of cancer and the significant resources needed for its management; many countries have put forward public responses to address this kind of challenge [7]. In the United States, a comprehensive national cancer control plan was established in the 1990s to mitigate the impact of cancer on the Population. The government of India launched the National Cancer Control Program (NCCP) in 1975 and revised the strategies from 1984 to 1985, which stressed primary prevention and early cancer detection [8].

Common cancer types vary by gender, with prostate, lung, colorectal, stomach, and liver cancers prevailing among men, while breast, colorectal, lung, cervical, and thyroid cancers predominate among women. Prostate, lung bronchus (from now on the lung), and colorectal cancers (CRCs) account for almost one-half (48%) of all incident cases in men, with prostate cancer alone accounting for 29% of diagnoses. Reports suggest that breast cancer, lung cancer, and colorectal cancer (CRC) make up 52% of all new cancer diagnoses in women, with breast cancer alone accounting for 31% of female cancer cases. In India, the burden of cancer is substantial and escalating, with projections indicating a rise from nearly one million new cases in 2012 to over 1.5 million by 2035 [9,10]. Notably, cancers of the oral cavity, uterus, and breast are prevalent in India, collectively representing approximately 4% of all cancer cases, as the data suggested [11].

In India, cancer-related mortality poses a significant concern for both citizens and the government, prompting the implementation of the National Program for Cancer, Diabetes, and Cardiovascular Diseases & Stroke (NPCDCS) to address such problems. This public health initiative is funded through the National Health Mission, emphasizing screening and early diagnosis at Community Health Centers and District Hospitals. At the same time, treatment is provided at regional Cancer Centers and tertiary care facilities. Despite efforts to address the cancer burden, challenges persist, particularly in low and middle-income countries like India. The main issues are limited health system capacity and disparities in access to quality care, which exacerbate the problem. Regional disparities further compound the challenge, with varying data availability hindering comprehensive understanding and intervention efforts. A detailed examination of cancer epidemiology is imperative for informing targeted strategies and policies [12,13]. However, the lack of comprehensive data on cancer patients, especially within the context of Eastern India, makes it more challenging to implement the policies at regional cancer centers and tertiary care facilities.

The number of gallbladder and head-and-neck cancers registered increased by 36% (between 2014 and 2015), 5% (between 2015 and 2016), 24% (between 2014 and 2015), and 4% (between 2015 and 2016), respectively. Carcinoma breast and cervix showed a decreasing trend, with registration falling to 13% (between 2015 and 2016) and 27% (between 2015 and 2016), respectively [14].

In light of these considerations, this study aims to elucidate the clinical and prevalence patterns of different cancers in multiple tertiary care cancer hospitals across Eastern India, focusing on patients from states such as Jharkhand, West Bengal, Bihar, and neighboring regions like Chhattisgarh. Through this study, we seek to contribute to a deeper understanding of cancer prevalence in the region and inform evidence-based interventions to mitigate its impact.

## Materials and methods

Study design

A descriptive cross-sectional retrospective study on various cancer types was conducted at the Tertiary Care Medical Teaching Cancer Hospital at the Department of Oncology affiliated with Rajendra Institute of Medical Sciences, Ranchi, in Eastern India. Data collected between July 2021 and December 2023 was utilised for the study.

Study population

This study included 1000 cancer patients whose data were obtained from the hospital's Electronic Health Records (EHR). Patients of either gender, on treatment, or newly diagnosed who visited the outpatient department of the hospital during the specified study period were enrolled. Terminally ill cancer patients and patients who did not visit the outpatient department of the studied site were excluded.

Data collection and statistical analysis

Socio-demographic details, clinical characteristics, treatment modalities, and patient-reported outcomes were noted for each participant from EHR data. However, the current study primarily focused on the prevalence of different types of cancer, categorized by gender and divided demographically by different zones for data calculation. Descriptive statistics, including mean, median, and percentage, were used to summarize the data. Data analysis was conducted using SPSS statistical software, version 20.0 (IBM Corp., Armonk, NY). A cross-sectional study design is generally used to assess the prevalence of a disease in a population, as the information is collected at a single point in time.

Ethical considerations

Approval for the study was obtained from the Institutional Ethics Committee at Rajendra Institute of Medical Sciences (IRC/MAR24/116) to ensure the protection of participants' rights and welfare before the commencement of the study. All procedures performed in the study involving human participants were in accordance with the ethical standards of the institutional research committee and with the 1964 Helsinki Declaration. We requested a waiver of written informed consent as the retrospective data collection was obtained from EHR data.

## Results

Age and gender distribution

Patients were further stratified by age and gender. Table 1 summarizes the distribution of various cancer types across different age groups. Notably, malignancies of the lip, oral cavity, and pharynx were most prevalent in the 41-60 age groups, while gastrointestinal tract cancers were prominent across all age groups.

**Table 1:** The distribution of various cancer types across different age groups CNS: Central nervous system

Types of Cancer	Age 0-20 years N (%)	Age 21-40 years N (%)	Age 41-60 years N (%)	Age 61-80 years N (%)	Age 81-Above N (%)	Total N (%)
Lip, oral cavity and pharynx	2 (1.25%)	56 (35%)	77 (48.12%)	25 (15.62%)	0 (0.0%)	160 (16.0%)
Gastrointestinal tract	1 (0.31%)	76 (24.28%)	155 (49.52%)	80 (25.55%)	1 (0.31%)	313 (31.3%)
Respiratory and intrathoracic organs	0 (0.0%)	7 (50%)	4 (28.57%)	3 (21.43%)	0 (0.0%)	14 (1.4%)
Bone and articular cartilage	3 (10.34%)	5 (17.24%)	20 (68.97%)	1 (3.44%)	0 (0.0%)	29 (2.9%)
Skin	0 (0.0%)	8 (33.33%)	9 (37.5%)	7 (29.17%)	0 (0.0%)	24 (2.4%)
Mesothelium and soft tissue	6 (22.22%)	8 (29.63%)	7 (25.93%)	5 (18.51%)	1 (3.70%)	27 (2.7%)
Breast		52 (26.26%)	119 (60.10%)	24 (12.12%)	1 (0.50%)	198 (19.8%)
Female genital organs	1 (1.44%)	20 (28.98%)	37 (53.62%)	9 (13.04%)	2 (2.90%)	69 (6.9%)
Male genital organs	0 (0.0%)	10 (47.62%)	4 (19.04%)	6 (28.57%)	1 (4.76%)	21 (2.1%)
Urinary tract	0 (0.0%)	1 (16.67%)	5 (83.33%)	0 (0.0%)	0 (0.0%)	6 (0.6%)
Eye, brain and other parts of CNS	0 (0.0%)	1 (100% )	0 (0.0%)	0 (0.0%)	0 (0.0%)	1 (0.1%)
Ill-defined, secondary and unspecified sites	2 (7.40%)	11 (40.70%)	9 (33.33%)	5 (18.51%)	0 (0.0%)	27 (2.7%)
Lymphoid, haematopoietic and related tissue	0 (0.0%)	3 (33.33%)	2 (22.22%)	4 (44.44%)	0 (0.0%)	9 (0.9%)
In situ neoplasms	0 (0.0%)	5 (25%)	7 (35%)	7 (35%)	1 (5%)	20 (2.0%)
Benign neoplasms	7 (10.14%)	21 (30.43%)	31 (44.93%)	10 (14.49%)	0 (0.0%)	69 (6.9%)
Neoplasms of uncertain or unknown behaviour	1 (14.28%)	3 (42.85%)	3 (42.86%)	0 (0.0%)	0 (0.0%)	7 (0.7%)
Aplastic and other anaemia’s	0 (0.0%)	0 (0.0%)	0 (0.0%)	1 (100%)	0 (0.0%)	1 (0.1%)
Total	25 (100.0%)	289 (100.0%)	490 (100.0%)	188 (100.0%)	8 (100.0%)	1000 (100.0%)

**Figure 1:**
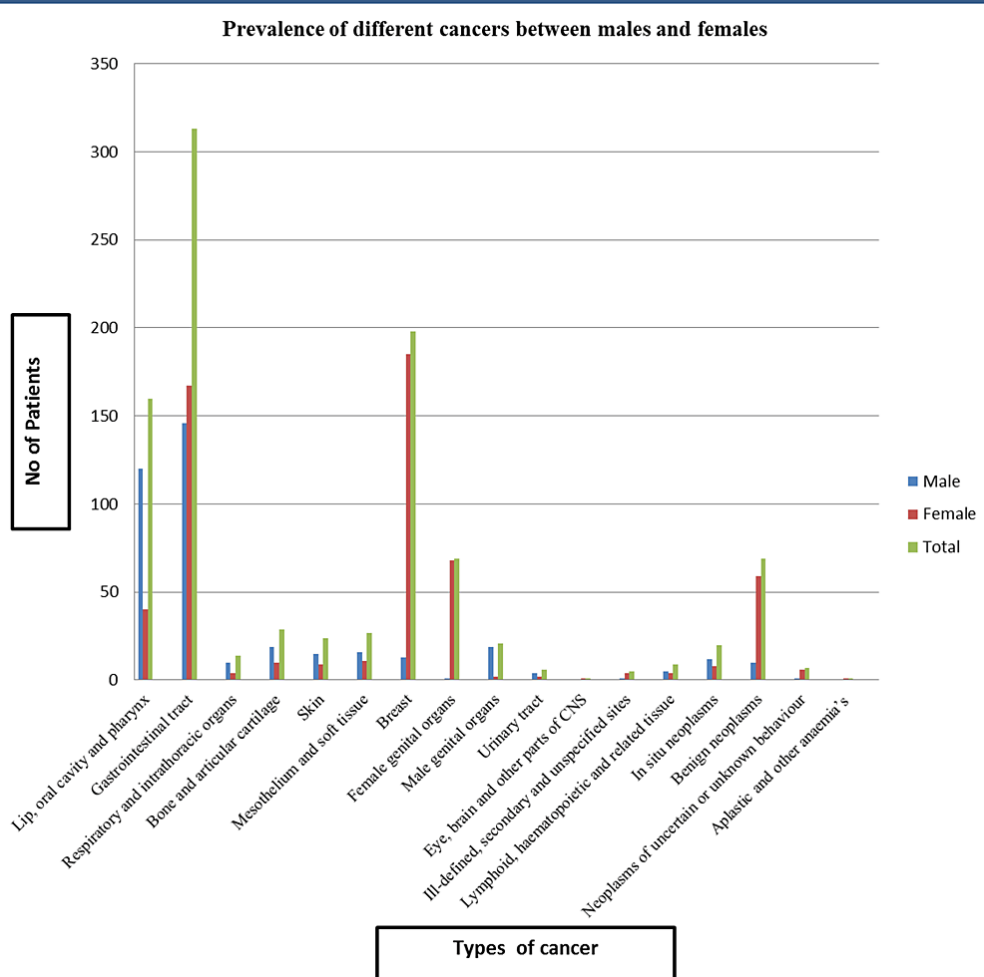
Represents the prevalence of different cancers between males and females

Table 2 and Figure 1 represent the prevalence of different cancers between males and females. Breast cancer emerged as the most common malignancy among females, whereas gastrointestinal tract cancers predominated among males. Overall, the most prevalent cancer site was the gastrointestinal tract organs, followed by breast cancer and lip, oral cavity, and pharynx carcinoma.

**Table 2:** Occurrences of different types of cancers in males and females CNS: Central nervous system

Type of cancers	Male N (%)	Female N (%)	Total N (%)
Lip, oral cavity and pharynx	120 (75.0%)	40 (25%)	160 (16.0%)
Gastrointestinal tract	146 (46.65%)	167 (53.35%)	313 (31.3%)
Respiratory and intrathoracic organs	10 (71.42%)	4 (28.57%)	14 (1.4%)
Bone and articular cartilage	19 (65.52%)	10 (34.48%)	29 (2.9%)
Skin	15 (62.50%)	9 (37.5%)	24 (2.4%)
Mesothelium and soft tissue	16 (59.26% )	11 (40.74%)	27 (2.7%)
Breast	13 (06.57%)	185 (93.43%)	198 (19.8%)
Female genital organs	1 (1.45%)	68 (98.55%)	69 (6.9%)
Male genital organs	19 (90.47%)	2 (09.50%)	21 (2.1%)
Urinary tract	4 (66.66%)	2 (33.33%)	6 (0.6%)
Eye, brain and other parts of CNS	0 (0.0%)	1 (100%)	1 (0.1%)
Ill-defined, secondary and unspecified sites	1 (20%)	4 (80%)	5 (0.5%)
Lymphoid, haematopoietic and related tissue	5 (55.56%)	4 (44.44%)	9 (0.9%)
In situ neoplasms	12 (60.00%)	8 (40.00%)	20 (2.0%)
Benign neoplasms	10 (14.49%)	59 (85.50%)	69 (6.9%)
Neoplasms of uncertain or unknown behaviour	1 (14.28%)	6 (85.71%)	7 (0.7%)
Aplastic and other anaemia’s	0 (0.0%)	1 (100%)	1 (0.1%)
Total	411 (100.0%)	589 (100.0%)	1000 (100.0%)

State-wise distribution

Table 3 outlines the prevalence of different cancers across various regions of states. North Chotanagpur exhibited the highest incidence of cancer cases, followed by South Chotanagpur. Gastrointestinal tract cancers were consistently prevalent across all states, with breast cancer being the second most common malignancy. Palamu recorded the lowest number of cancer cases among the surveyed states.

**Table 3:** Prevalence of different cancers in various states

Type of cancers	Palamu division N (%)	North Chotanagpur N (%)	South Chotanagpur N (%)	Kolhan N (%)	Santhal pargana N (%)	West Bengal N (%)	Bihar N (%)	Other states N (%)	Total N (%)
Lip, oral cavity and pharynx	9 (05.62%)	53 (33.12%)	39 (24.373%)	7 (4.37%)	23 (14.38%)	11 (6.88%)	14 (8.75%)	4 (2.5%)	160 (16.0%)
Gastrointestinal tract	10 (03.19%)	121 (38.66%)	77 (24.60%)	18 (05.75%)	43 (13.73%)	30 (09.58%)	14 (04.47%)	0 (0.0%)	313 (31.3%)
Respiratory and intrathoracic organs	1 (07.14%)	6 (42.86%)	2 (14.86%)	0 (0.0%)	2 (14.86%)	3 (21.43%)	0 (0.0%)	0 (0.0%)	14 (1.4%)
Bone and articular cartilage	3 (10.34%)	16 (55.17%)	4 (13.79%)	0 (0.0%)	2 (6.89%)	4 (13.79%)	0 (0.0%)	0 (0.0%)	29 (2.9%)
Skin	6 (25.00%)	7 (29.17%)	6 (25.00%)	0 (0.0%)	2 (8.33%)	3 (12.5%)	0 (0.0%)	0 (0.0%)	24 (2.4%)
Mesothelium and soft tissue	1 (3.7%)	11 (40.74%)	8 (29.63%)	1 (3.70%)	5 (18.51%)	0 (0.0%)	1 (3.7%)	0 (0.0%)	27 (2.7%)
Breast	4 (2.02%)	96 (48.48%)	56 (28.28%)	0 (0.0%)	16 (8.08%)	7 (3.53%)	16 (8.08%)	3 (1.51%)	198 (19.8%)
Female genital organs	5 (7.24%)	29 (42.02%)	19 (27.54%)	2 (2.89%)	7 (10.14%)	3 ( 4.35%)	4 (5.80%)	0 (0.0%)	69 (6.9%)
Male genital organs	1 (4.76%)	11 (52.38%)	6 (28.57%)	2 (09.52%)	0 (0.0%)	0 (0.0%)	1 (4.76%)	0 (0.0%)	21 (2.1%)
Urinary tract	1 (16.67%)	3 (50%)	1 (16.67%)	1 (2.6%)	0 (0.0%)	0 (0.0%)	0 (0.0%)	0 (0.0%)	6 (0.6%)
Eye, brain and other parts of CNS	0 (0.0%)	0 (0.0%)	0 (0.0%)	1 (100%)	0 (0.0%)	0 (0.0%)	0 (0.0%)	0 (0.0%)	1 (0.1%)
Ill-defined, secondary and unspecified sites	1 (03.70%)	9 (33.33%)	10 (37.04%)	1 (03.70%)	3 (11.11%)	2 (7.40%)	1 (3.70%)	0 (0.0%)	27 (2.7%)
Lymphoid, haematopoietic and related tissue	2 (22.22%)	4 (44.44%)	2 (22.22%)	1 (11.11%)	0 (0.0%)	0 (0.0%)	0 (0.0%)	0 (0.0%)	9 (0.9%)
In situ neoplasms	2 (10.00%)	6 (30.00%)	6 (30.00%)	1 (05.00%)	1 (05.00%)	2 (10%)	2 (10%)	0 (0.0%)	20 (2.0%)
Benign neoplasms	2 (04.20%)	24 (05.90%)	22 (08.50%)	3 (07.70%)	7 (06.30%)	3 (4.4%)	8 (13.10%)	0 (0.0%)	69 (6.9%)
Neoplasms of uncertain or unknown behaviour	0 (0.0%)	4 (57.14%)	2 (28.57%)	0 (0.0%)	1 (14.28%)	0 (0.0%)	0 (0.0%)	0 (0.0%)	7 (0.7%)
Aplastic and other anaemia’s	0 (0.0%)	1 (100%)	0 (0.0%)	0 (0.0%)	0 (0.0%)	0 (0.0%)	0 (0.0%)	0 (0.0%)	1 (0.1%)
TOTAL	48 (100.0%)	405 (100.0%)	260 (100.0%)	39 (100.0%)	112 (100.0%)	68 (100.0%)	61 (100.0%)	7 (100.0%)	1000 (100.0%)

Patients were categorized according to their geographic zones of origin, including Palamu division (n=48), North Chotanagpur division (n=405), South Chotanagpur division (n=260), Kolhan division (n=39), Santhal Pargana division (n=112), West Bengal (n=68), Bihar (n=61), and other states.

## Discussion

This study presents the prevalence patterns of common cancer types across tertiary care cancer hospitals in Eastern India, focusing on regions such as Jharkhand, West Bengal, Bihar, and others. The findings of this research hold considerable clinical significance for several reasons. Firstly, it offers valuable insights into the prevalence of various cancers, aiding in implementing targeted interventions to address the specific needs of these areas. Secondly, it provides clinicians with essential information regarding the distribution of cancers across different age groups and genders, enabling more informed decisions regarding diagnostic approaches, treatment plans, and follow-up care. Thirdly, the observed variations in cancer prevalence among different states can inform resource allocation strategies, ensuring adequate infrastructure, equipment, and healthcare professionals are available in regions with higher cancer burdens.

As per the result of the current study, higher susceptibility to cancer is found in the 41-60-year-old age groups, followed by the 21-40-year-old age groups, highlighting the necessity for age-specific cancer prevention strategies and regular health check-ups for these cohorts. Additionally, while the lower incidence of cancer in the population aged 81 and above may be attributed to factors such as decreased exposure to risk factors or natural decline in cell division, attention is still warranted to ensure access to appropriate cancer care and supportive services for this age group.

Regional variations reveal specific areas with higher cancer burdens, assisting healthcare providers and policymakers in resource allocation and examining gender prevalence differences. Our findings indicate a higher prevalence of cancer in females compared to males, with breast cancer being the most common among females and cancer of the gastrointestinal tract predominating among males. Nikbakhsh and colleagues conducted a similar study in Babol, Iran, which involved 150 patients recently diagnosed with various cancers [15]. Their findings revealed that 52% of the 59.04 ± 14.34 years among the patients. This suggests that our study's findings aligned with this group's previous findings. These gender-specific patterns underscore the importance of tailored approaches in cancer prevention. Trends in cancer incidence and overall cancer incidence rates, which reflect both patterns in behaviors associated with cancer risk and changes in medical practice, such as the use of cancer screening tests surge in incidence among males during the early 1990s, can be attributed to heightened detection of asymptomatic prostate cancer, driven by the widespread adoption of prostate-specific antigen (PSA) testing among previously unscreened individuals. Subsequently, cancer incidence in men generally declined until around 2013 and stabilized through 2019. In contrast, the incidence rate in women remained relatively stable until the mid-1980s but has shown a slow increase of less than 0.5% per year. Consequently, the sex gap in cancer incidence is gradually narrowing, with the male-to-female incidence rate ratio decreasing from 1.59 (95% confidence interval (CI), 1.57-1.61) in 1992 to 1.14 (95% CI, 1.14-1.15) in 2019, these all shows the recent trends in cancer incidence rates, screening, and treatment [15,16].

Among females, breast cancer (31.4%) was the most common, followed by malignancies of the gastrointestinal tract (28.4%). For males, cancer of the gastrointestinal tract (35.5%) was the most prevalent, followed by lip, oral cavity, and pharynx carcinoma (29.2%). These gender-specific patterns highlight the importance of tailored approaches in cancer prevention, screening, and treatment. Strategies with increasing awareness programs such as promoting breast self-examinations and mammograms for females and addressing the risk factors such as tobacco use among males can significantly contribute to reducing the burden of these cancers. The findings of this study were the results of the study conducted by Mishra and co-workers in 2006 [16]. They recruited 38 asymptomatic or minimally symptomatic cancer patients and found that the maximum number of participants (63%) had upper aero-digestive tract (UADT) malignancies. However, Saranath and group [17] have reported that the most common cancer in men was lip and oral cavity carcinoma in their study.

Interestingly, women have exhibited breast carcinoma as the most prevalent type. The findings supported this study about females but were in contrast with the reference to males. This might be because our study needed a nationally representative sample and region. Berihun and colleagues surveyed at Gondar University Hospital, where they observed that most cancer patients (31.2%) had blood cancers [18]. The discrepancy in these findings could be attributed to differences in the countries or geographical locations where the study had been conducted and variations in the estimated sample sizes and the types of cancer patients included in their respective study settings.

Furthermore, our study identifies North Chotanagpur and South Chotanagpur as regions with the highest cancer prevalence, signaling the need for targeted interventions and healthcare resources in these areas. Additionally, this calls for urgent attention and focused efforts to address the factors contributing to the high cancer burden in these regions. Identifying and targeting specific risk factors, improving access to cancer screening and early detection services, and enhancing treatment facilities could help alleviate cancer's impact in these areas. Further, the most prominent cancer across all regions was cancer of the gastrointestinal tract organs (31.3%), followed by breast cancer (19.8%). This indicates the need for targeted prevention and control strategies for these types of cancer. The association between gastric cancer and tobacco smoking has been observed in various epidemiological studies. In India, not only tobacco smoking but also tobacco chewing is highly prevalent. Tobacco is used in various forms, such as hukka, snuff, bidis, cigarettes, taibur, meiziol, etc. About 229,392,725 adult males and 11,908,517 adult females are estimated to use tobacco in India. Tobacco chewing and gutkha are pretty prevalent in Eastern India [19-21].

Implementing comprehensive tobacco control programs to address the high prevalence of tobacco chewing, a common risk factor for gastrointestinal cancers and head and neck cancer, is essential [22-24]. Additionally, promoting breast cancer awareness, regular screenings, and access to quality care services can contribute to early detection and improved treatment outcomes for breast cancer [23]. Additionally, urgent attention and focused efforts are required to address the factors contributing to the high cancer burden in these regions.

Study limitations

Clinical information includes cancer stage, duration since diagnosis, treatment modalities, and outcomes, while non-clinical details include habits, other co-morbidities or physical illness, family history, and socio-economic status. This information could provide a more comprehensive understanding of the disease burden.

## Conclusions

In conclusion, this study provides significance for understanding cancer prevalence and distribution across different regions, age groups, and genders. The findings provide a foundation for evidence-based decision-making, resource allocation, and the development of targeted interventions. Continuous monitoring of cancer trends, collaborative efforts between stakeholders, and further research are essential to address cancer's challenges and reduce its burden on society. Additional research and collaborations are warranted to validate these findings and effectively implement evidence-based interventions to combat cancer.
